# Policy: WHO/ILSI Affiliation Sustained

**DOI:** 10.1289/ehp.114-a521a

**Published:** 2006-09

**Authors:** Tim Lougheed

The International Life Sciences Institute (ILSI) is set to be honored for its Physical Activity and Nutrition program as part of the September 2006 National Congress on Accelerating Improvement in Childhood Obesity in Washington, DC. The program is being singled out for its innovative approaches to educating children, parents, and caregivers about managing child obesity. However, such praise contrasts sharply with criticism that was leveled at ILSI just months ago.

At a 27 January 2006 meeting in Geneva, the WHO Executive Board reviewed ILSI’s NGO status, along with that of several other NGOs, as part of the WHO’s standard three-year review cycle. The National Resources Defense Council, an environmental action group, was calling for the WHO to sever its relationship with ILSI, based on the fact that ILSI’s membership includes representatives of major multinational corporations with a vested interest in public health matters.

The board ultimately decided to maintain official relations with ILSI, describing its role as “a knowledge resource base for the application of leading-edge science and knowledge transfer in the fields of food and chemical safety.” ILSI is therefore still permitted to attend WHO governing body meetings and make statements at these meetings. Although this presence does not come with voting privileges, it affords such an organization valuable “insider” status. However, this latest decision by the Executive Board does exclude any collaboration by ILSI on normative activities, defined as “setting microbiological or chemical standards for food and water.”

According to Harvey Anderson, who chairs ILSI’s Board of Trustees, the organization has never taken part in such activities anyway. While its strongest critics accuse ILSI of working behind the scenes to present information that would shape public policy to corporate advantage, Anderson points out that ILSI functions according to a nonprofit charter bound by U.S. regulations, with a board dominated by members not affiliated with any vested interest, who would readily vote down such lobbying.

“ILSI is overrun by lawyers making damn sure everybody holds to the charter,” he explains, noting that representatives like himself—a nutritional sciences professor at the University of Toronto—are unpaid volunteers devoting a great deal of time and energy to what they regard as a research endeavour.

“ILSI has gathered some of the top scientists from around the world to work with industry and work with government and work with their colleagues on issues that need this kind of cooperative approach,” says Anderson. “We publish our research and reports in journals that require peer review by their standards and by scientists of their choice.”

Observers such as Jennifer Sass, a scientist with the Natural Resources Defense Council, insist that this approach nevertheless yields skewed science. Her group drafted a letter of protest that was circulated by hand at the Geneva meeting, citing examples of ILSI’s undue influence in shaping the conclusions of scientific studies on issues such as the role of sugar in diet and the designation of cancer-causing agents.

“Their documents come out in one direction, which is always somewhere between ‘everything’s okay’ and ‘we need more study,’” she says, referring to ILSI’s participation in a 1998 study that denied finding evidence of a direct link between sugar intake and lifestyle-related diseases. According to Sass, that conclusion conflicts with the results from a 1990 WHO study group, which recommended health policy targets that reflect the increased risks of chronic diseases caused by dietary patterns that include high sugar intake.

“They never come out as ‘this is a big problem and we should do something about it,’” says Sass, adding that governments can perpetuate a misrepresentation of this research by condoning ILSI’s approach.

Similar complaints voiced in the 1990s were followed by a 2001 report by the WHO Tobacco-Free Initiative on how member tobacco companies had used ILSI to muster seemingly unbiased scientific opinions aimed at quashing WHO tobacco control efforts. ILSI vehemently denied these allegations and, Anderson says, worked with the WHO to provide assurances of transparency about how ILSI operates.

Derek Yach, now head of the Rockefeller Foundation’s Program on Global Health, led that WHO initiative and closely watched ILSI’s response. “Part of the problem lies with the UN agencies themselves, or in this case the WHO,” he concludes, noting that these bodies have yet to take many of the protective measures recommended by the report. “You can blame the corporations,” he says, “but you also need to place blame on not having very clear, very transparent review of conflict-of-interest procedures internally.”

